# Supplementary material for: Anticonvulsant Potential and Toxicological Profile of Verbesina persicifolia Leaf Extracts: Evaluation in Zebrafish Seizure and Artemia salina Toxicity Models
Source: Plants (Basel). 2025 Apr 1;14(7):1078. doi: 10.3390/plants14071078 (PMC11991391; doi:10.3390/plants14071078)
Supplement: Supplementary file 1 [file plants-14-01078-s001.zip › plants-3514534-supplementary.pdf]

Supporting Information

Anticonvulsant Potential and Toxicological Profile of *Verbesina persicifolia* Leaf Extracts: Evaluation in Zebrafish Seizure and Artemia salina Toxicity Models

Carlos Alberto López-Rosas [1], Santiago González-Periañez [1], Tushar Janardan Pawar [2], Jorge Zurutuza-Lorméndez [1], Fernando Rafael Ramos-Morales [1], José Luís Olivares-Romero[2], Margarita Virginia Saavedra Vélez [1,*] and Fabiola Hernandez-Rosas [3,*]

[1] Instituto de Química Aplicada, Universidad Veracruzana, Luis Castelazo Ayala s/n, Col. Industrial Animas, 91190 Xalapa-Enríquez, Veracruz, México.

[2] Red de Estudios Moleculares Avanzados, Campus III, Instituto de Ecología A. C., Carretera Antigua a Coatepec 351, Xalapa, 91073, Veracruz, Mexico.

[3] Centro de Investigación, Universidad Anahuac Querétaro, El Marqués, Querétaro 76246, Mexico.

Correspondence: (M.V.S.-V) msaavedra@uv.mx

and (F.H.-R) fabiola.hernandezro@anahuac.mx

Index

1. **Characteristics of *V. persicifolia*** **S2**
2. **Collection and Extraction of *V. persicifolia*** **S4**
3. **Phytochemical Analysis of *V. persicifolia*** **S8**
4. **Experimental Procedures** **S10**
5. **Statistical Analysis** **S13**
6. **References** **S33**

1. Characteristics of *V. persicifolia*

V. persicifolia is a perennial shrub belonging to the Asteraceae family, characterized by its elongated serrated leaves and bright yellow capitulum-shaped flowers. The leaves can grow up to 14 cm in length, exhibiting a lanceolate shape with serrated edges (Figure A). The flowers are arranged in dense, terminal clusters, forming numerous capitula, which are typical of species within the Verbesina genus (Figure S1).

This species is naturally distributed in warm and semi-warm climates, thriving at altitudes between 200 and 1850 meters above sea level (m.a.s.l.). It is commonly associated with tropical dry forests, sub-deciduous forests, and xerophytic shrublands, where it plays an important role in local biodiversity and ecological interactions [1].

1.1. Geographical Distribution & Habitat

V. persicifolia is endemic to Mexico, predominantly found in the states of Puebla, Tlaxcala, and Veracruz. It grows in well-drained soils with moderate organic matter and is often found along roadsides, forest edges, and disturbed habitats. The plant is drought-tolerant and adapts well to seasonal variations, making it a resilient species within its native range.

1.2. Traditional & Ethnobotanical Uses

Ethnobotanical studies suggest that species of the *Verbesina* genus have been traditionally used in Mexican herbal medicine for their anti-inflammatory, wound-healing, and neuroactive properties. While specific reports on *V. persicifolia* remain limited, related species such as *Verbesina crocata* and *Verbesina encelioides* have demonstrated medicinal applications, particularly for treating skin conditions, digestive disorders, and neurological ailments [2]. These traditional applications highlight the potential pharmacological value of *V. persicifolia* and justify further investigation into its bioactive compounds.

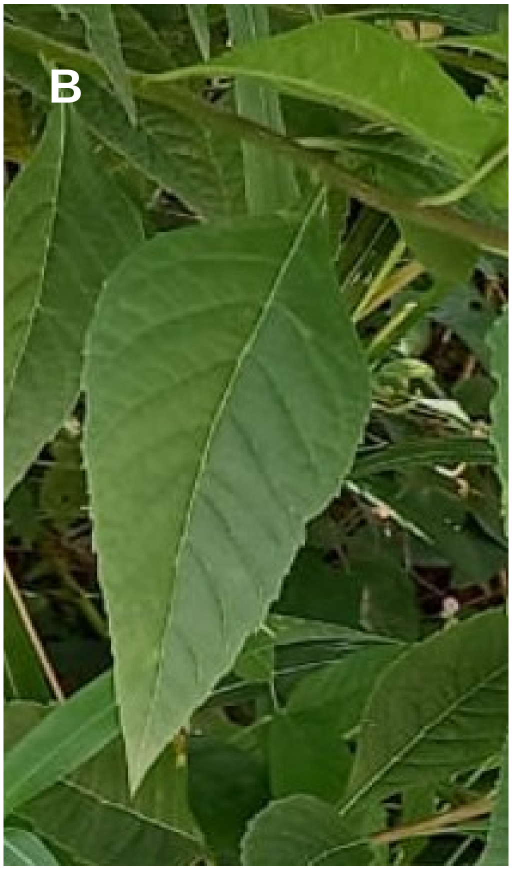

Figure S1. Morphological characteristics of *V. persicifolia*. **(A)** Shrub structure of *V. persicifolia*, **(B)** Elongated serrated-edged leaves, **(C)** Yellow capitulum-shaped flowers. *(Photographs by Carlos Alberto López Rosas).*

2. Collection and Extraction of *V. persicifolia*

The collection of *V. persicifolia* leaves is conducted annually during the last week of September, coinciding with the plant's flowering period (Figure S2). This seasonal selection ensures optimal phytochemical composition, as secondary metabolite production is often highest during flowering.

2.1. Plant Collection and Selection

The collection site is recorded each year using GPS coordinates to ensure that future samplings occur at the same ecological location. The selected leaves are manually inspected, and only healthy, undamaged specimens are retained for extraction. Leaves that are yellowed, dry, insect-damaged, or otherwise compromised are excluded to maintain consistency in extraction quality (Figure S3).

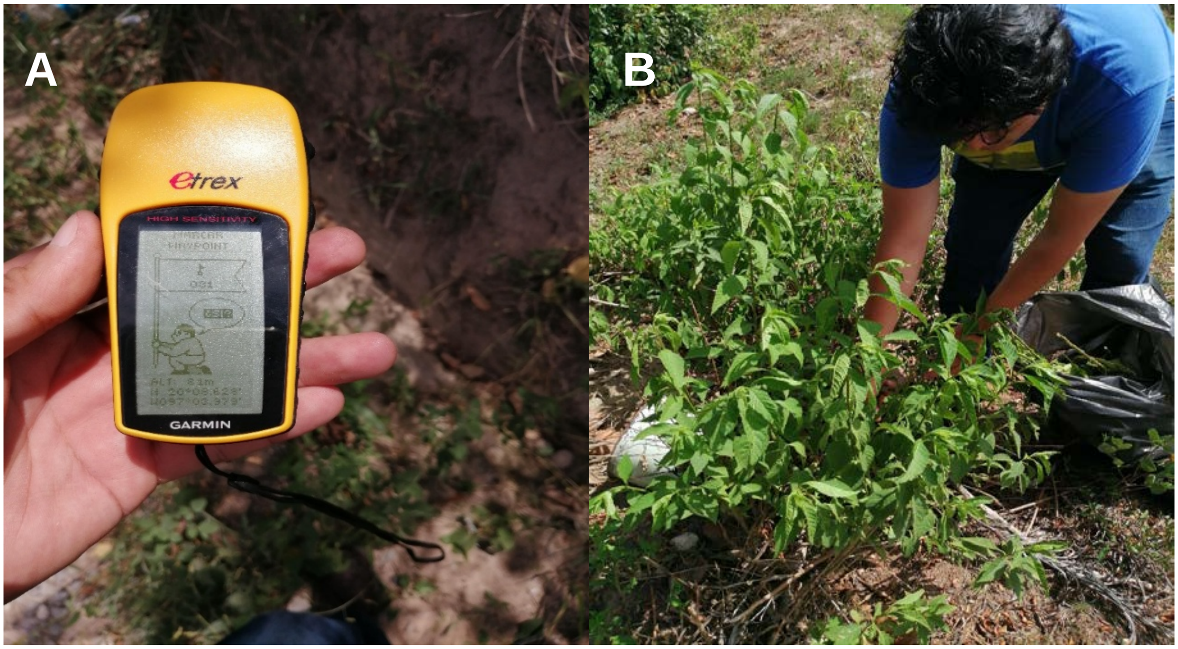

Figure S2. (A) Recordingof GPS coordinates to enable precise relocation in future collections. (B) Selection of plant material. *(Photographs by Carlos Alberto López Rosas).*

Figure S3. Leaves placed on absorbent material to facilitate aeration and drying. *(Photographs by Carlos Alberto López Rosas)*

2.2. Drying and Preparation of Plant Material

The selected leaves are air-dried in the shade to prevent photodegradation of bioactive compounds. Leaves are arranged in a well-ventilated environment using absorbent material to facilitate even drying (Figure S3). Once fully dried, the leaves are ground into a fine powder to increase the surface area for extraction (Figure S4).

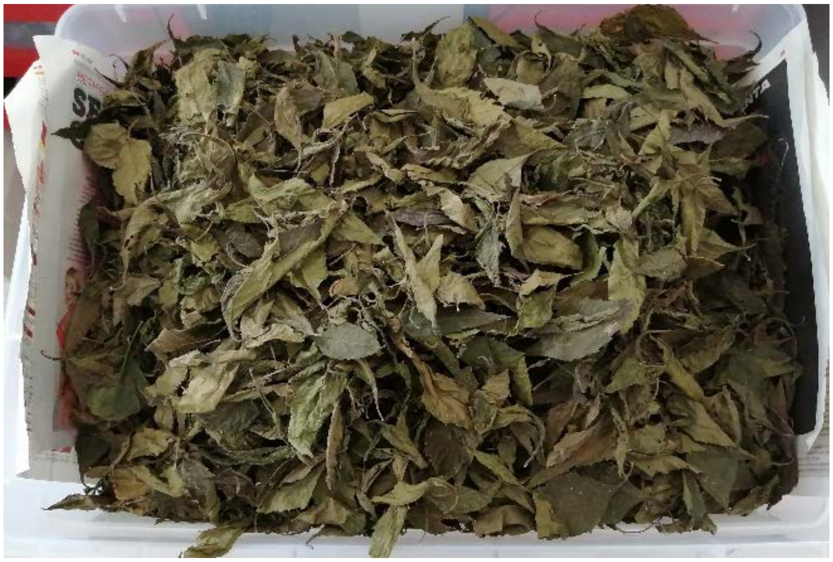

Figure S4. Dried *V. persicifolia* leaves before grinding. *(Photographs by Carlos Alberto López Rosas)*

2.3. Maceration Process

The powdered leaves are placed into amber glass containers to protect them from light-induced degradation. The material is subjected to maceration in methanol for 21 days under ambient conditions, allowing efficient extraction of secondary metabolites (Figure S5).

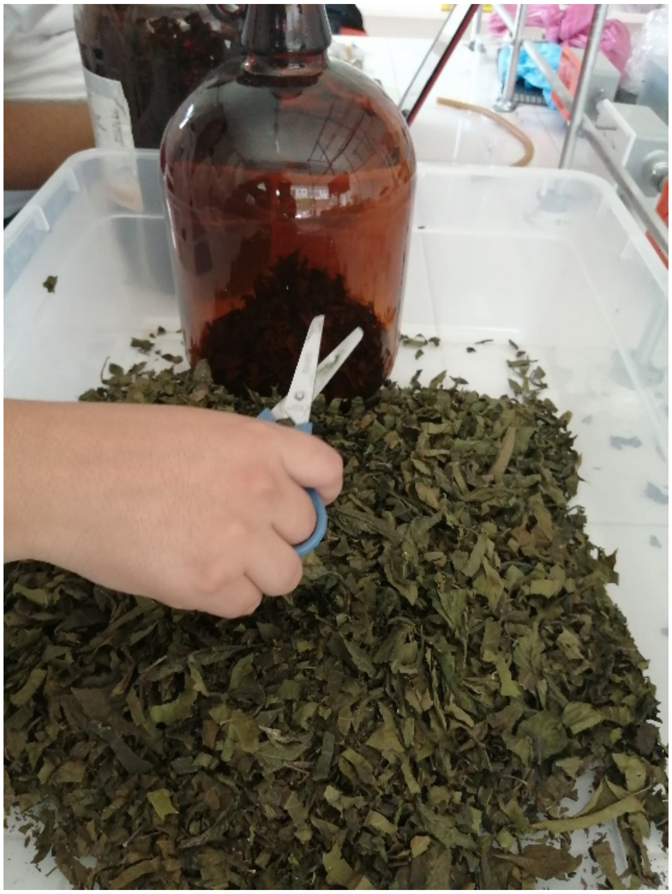

Figure S5. Amber glass flasks filled with dried plant material for maceration. *(Photographs by Carlos Alberto López Rosas)*

2.4. Methanolic Extract Preparation

After 21 days of maceration, the extract is filtered and concentrated under reduced pressure to remove excess solvent. The resulting crude extract undergoes evaporation in an oven at 40°C to eliminate residual solvent before fractionation (Figure S6). This process ensures that the extracts are free from excess methanol while preserving thermally stable bioactive compounds.

Figure S6. (A) Methanolic extract obtained after 21 days of maceration (third collection, 2021). (B) Concentrated extract after drying in an oven at 40°C. *(Photographs by Carlos Alberto López Rosas)*

2.5. Fractionation and Final Powder Preparation

The concentrated crude methanolic extract is further processed through fractionation using organic solvents. Sequential partitioning is performed with:

- Hexane (non-polar compounds)
- Dichloromethane (semi-polar compounds)
- Ethyl acetate (polar compounds)
- Water (highly polar compounds)

Each fraction is subjected to solvent evaporation under reduced pressure, yielding semi-solid residues. The remaining solvent traces are removed by placing the fractions in an oven at 40°C until a completely dry powder is obtained.

The final powders are stored in vacuum-sealed containers at 4°C to preserve their stability and prevent degradation. These dry extracts are later used for phytochemical analysis and biological assays.

Methodology adapted from [3,4]

3. Phytochemical Analysis of *V. persicifolia*

Phytochemical determinations were performed to qualitatively identify the secondary metabolites present in the different leaf extracts of *V. persicifolia*. The identification of bioactive compounds was conducted using thin-layer chromatography (TLC), with specific visualization reagents applied to detect different classes of secondary metabolites.

3.1. Thin-Layer Chromatography (TLC) Analysis

TLC was employed to separate and identify phytochemicals in different solvent fractions, allowing for qualitative screening of bioactive compounds. Extracts were applied to silica gel TLC plates and developed in appropriate solvent systems to achieve optimal separation of metabolites. The plates were then treated with specific visualization reagents to detect compound classes based on colorimetric reactions.

TLC Analysis of Non-Polar Extracts

The hexane and dichloromethane fractions were analyzed under a 95:5 dichloromethane/methanol mobile phase to enhance the separation of lipophilic compounds, including essential oils, steroids, and alkaloids. Various visualization reagents were used to detect functional groups:

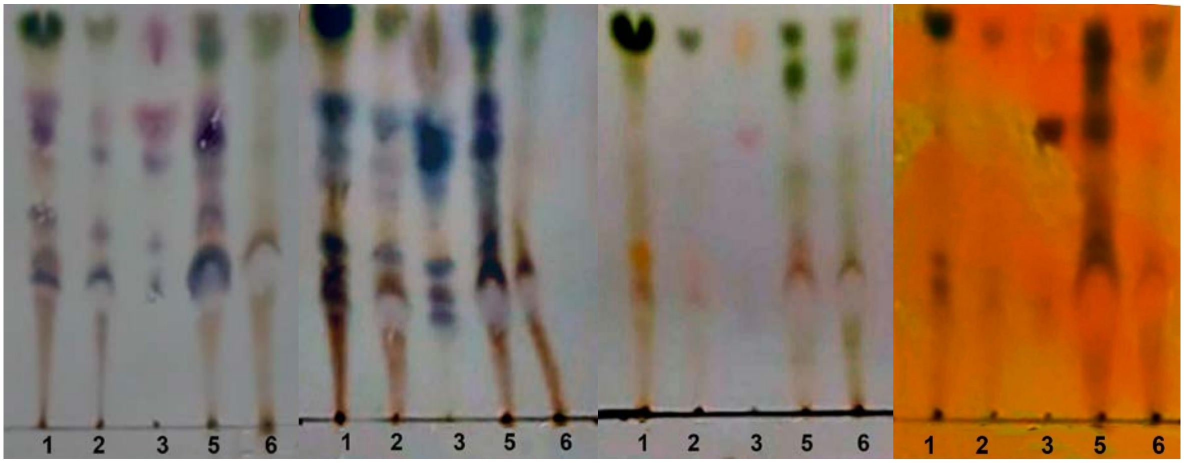

Figure S7. TLC analysis of hexane and dichloromethane fractions. (I) Ethyl acetate fraction. (II) Dichloromethane fraction. (III) Hexane fraction. Visualization reagents used: I) Cobalt chloride: General metabolite detection. II) Vanillin: Detection of phenols and steroids. III) Aluminum chloride: Detection of flavonoids. IV) Dragendorff reagent: Detection of alkaloids.

TLC Analysis of Polar Extracts

The methanol and aqueous fractions were analyzed using an 8:2 ethyl acetate/methanol mobile phase, which facilitated the separation of polar compounds, including flavonoids, tannins, and phenolic acids.

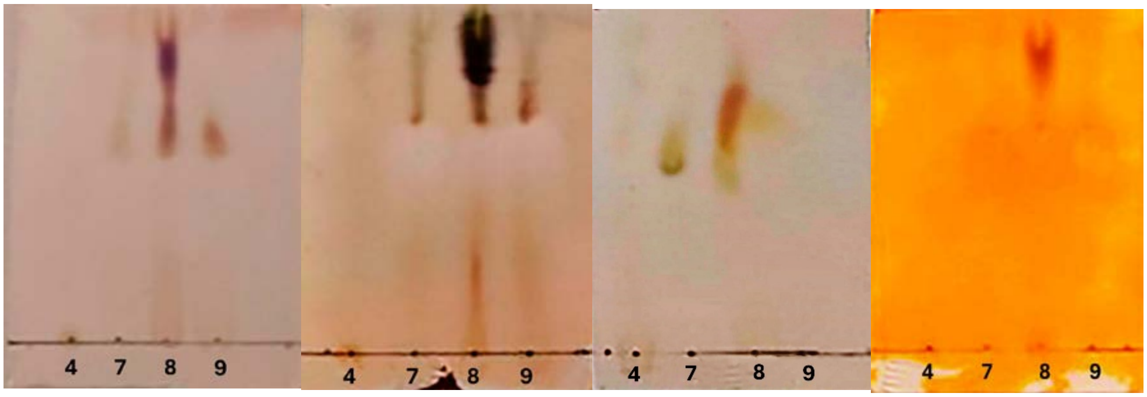

Figure S8. TLC analysis of methanol and aqueous fractions. (IV) Methanol fraction. (VII) Aqueous fraction. (VIII) Methanol partition. (IX) Ethyl acetate partition. Reagents applied for detection: I) Vanillin-sulfuric acid: Identifies terpenoids and phenolic compounds. II) Ferric chloride: Detects tannins and phenolic acids. III) Aluminum chloride: Confirms the presence of flavonoids.

3.2. ^{1}H NMR Analysis

To further characterize the secondary metabolites, present in V. persicifolia, 1H NMR analysis was performed on the hexane, dichloromethane, and ethyl acetate extracts. Figure S9 presents the recorded 1H NMR spectrum, which reveals distinct signals corresponding to flavonoid derivatives and eudesmane sesquiterpenes, two major classes of natural products previously reported in Verbesina species.

In the aromatic region (7.6–7.4 ppm, 6.4–6.5 ppm), multiple proton signals indicate the presence of hydroxylated or methoxylated flavonols, suggesting the occurrence of flavonoid-based structures. The multiplets in the glycosidic region (3.0–5.5 ppm) further indicate the presence of flavonoid glycosides, such as rutin or quercetin derivatives, although definitive identification requires further 2D NMR (HSQC, HMBC) and mass spectrometry.

In the aliphatic region (0.9–1.5 ppm), multiple methyl group signals are evident, which are characteristic of eudesmane sesquiterpenes. This aligns with previous phytochemical reports describing sesquiterpene lactones as key constituents of V. persicifolia.

While the observed NMR spectral features strongly suggest the co-occurrence of flavonoid derivatives and eudesmane sesquiterpenes, additional structural confirmation is required to determine the precise nature of the identified metabolites. The spectral data in Figure S9 serve as a fundamental analytical reference for future studies on the chemical composition of V. persicifolia.

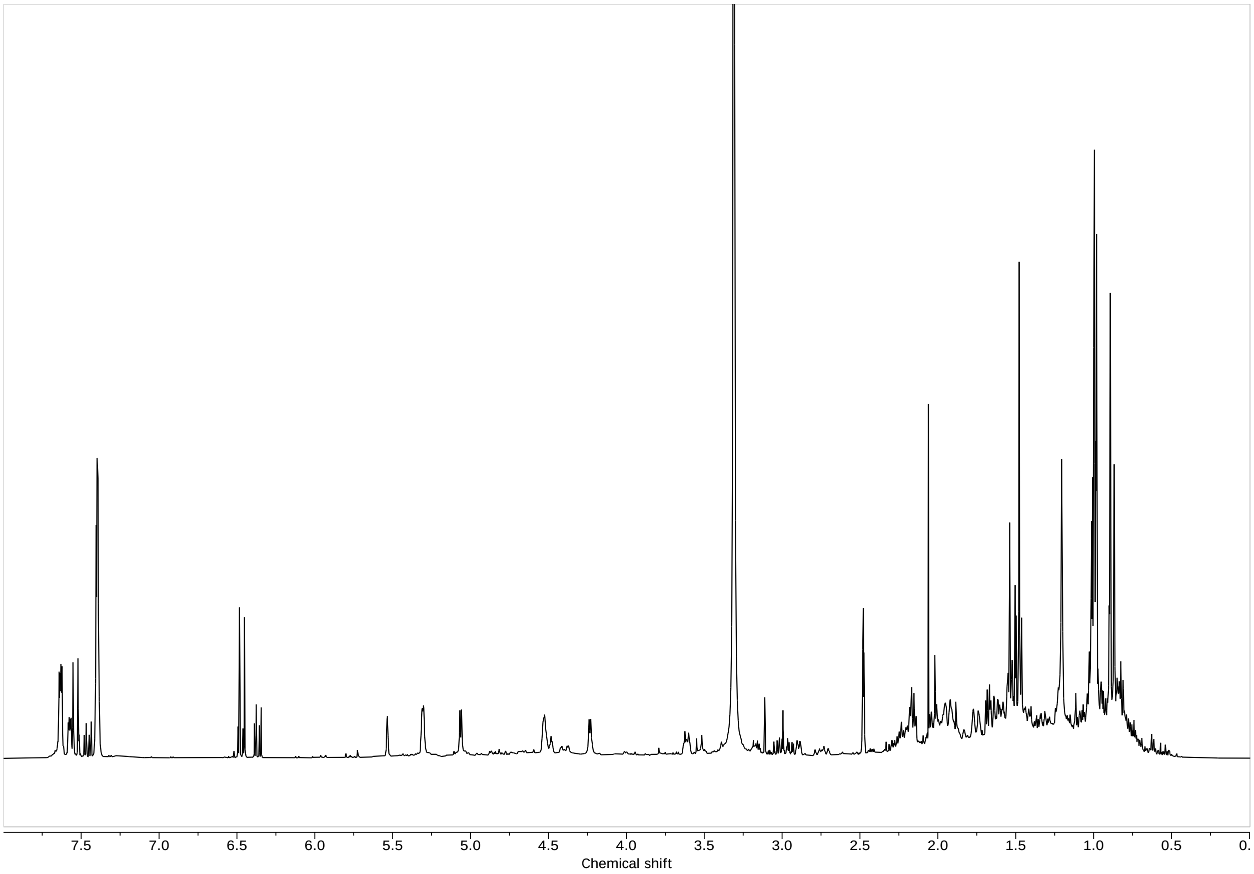

Figure S9. *^{1}H NMR spectrum of* Verbesina persicifolia *extracts (500 MHz, $CDCl_3$).* The spectrum exhibits signals corresponding to flavonoid derivatives and eudesmane sesquiterpenes, as suggested by the characteristic peaks in the aromatic (7.6–7.4 ppm, 6.4–6.5 ppm), glycosidic (3.0–5.5 ppm), and aliphatic (0.9–1.5 ppm) regions.

4. Experimental Procedures

This section provides detailed descriptions of the methodologies used to assess the **toxicological profile and anticonvulsant activity** of *V. persicifolia* extracts. These procedures were adapted from validated protocols to ensure experimental reproducibility.

4.1. Toxicological Evaluation in *Artemia salina*

The toxicity of *V. persicifolia* extracts was evaluated using the **brine shrimp lethality assay (BSLA)** with ***Artemia salina*** **nauplii**. The procedure followed standard protocols to assess **acute toxicity**, providing an estimate of the median lethal concentration (**LC_{50}**) for each extract.

Hatching & Maintenance of *A. salina*

A. salina cysts (350 mg) were incubated in 2 L of artificial seawater (3% NaCl solution) under controlled conditions:

- **Aeration:** Constant
- **Temperature:** 24–29°C
- **Light Exposure:** Continuous

After 48 hours, hatched nauplii were collected and transferred to experimental test wells.

Exposure to Extracts & Toxicity Assessment

Nauplii were exposed to various concentrations of *V. persicifolia* methanolic extracts: 1, 0.5, 0.25, 0.1, and 0.05 mg/mL (prepared in 3% saline solution).

After 24 hours of exposure, the number of surviving nauplii was recorded, and mortality rates were calculated.

The LC_{50} values were determined using Probit analysis with 95% confidence intervals, performed in IBM SPSS Statistics 29.

4.2. Anticonvulsant Activity in Zebrafish Model

The anticonvulsant potential of *V. persicifolia* extracts was evaluated using a pentylenetetrazol (PTZ)-induced seizure model in adult zebrafish (*Danio rerio*). This model allowed the assessment of extract efficacy based on seizure latency, severity, and survival rates.

Pre-Treatment & Dosing

Zebrafish were pre-treated by immersion in tanks containing different extract concentrations for 30 minutes before seizure induction.

Methanolic extracts were tested at 1, 10, 100, and 1000 µg/mL, while partitioned fractions were evaluated at 0.1, 1, 10, and 100 µg/mL.

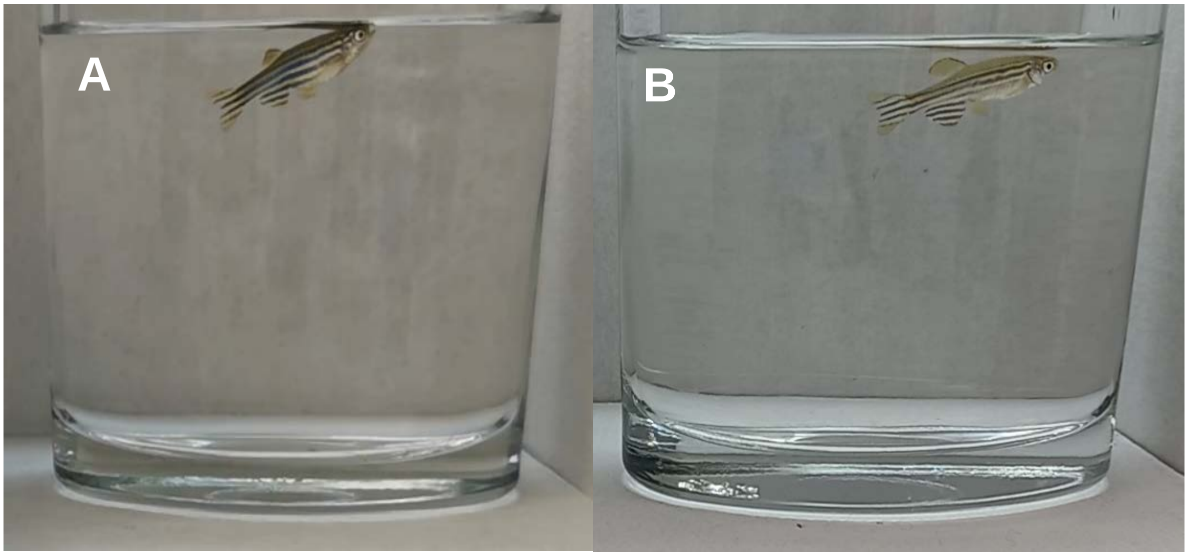

Figure S10. Zebrafish (*Danio rerio*) placed in **250 mL tanks containing PTZ** for the pharmacological evaluation of *V. persicifolia* extracts. **(A)** Zebrafish in normal conditions before PTZ exposure. **(B)** Zebrafish displaying altered behavior after PTZ exposure. *(Photographs by Carlos Alberto López Rosas).*

Seizure Induction & Behavioral Analysis

Following pre-treatment, zebrafish were transferred to a 10 mM PTZ solution to induce seizures.

Behavioral responses were recorded and classified into three distinct seizure stages:

- **Stage I:** Increased swimming activity
- **Stage II:** Whirlpool swimming

- **Stage III:** Clonus-like convulsions with loss of posture

Survival rates were monitored post-exposure to assess potential neuroprotective effects of the extracts.

This protocol was adapted from [5,6].

4.3. Co-administration with Pharmacological Controls

To evaluate potential synergistic anticonvulsant effects, the hexane fractions of *V. persicifolia*, which exhibited significant activity in the zebrafish model, were co-administered with diazepam and sodium valproate.

Pharmacological Control Preparation

Diazepam was dissolved in methanol, and sodium valproate was dissolved in ethyl acetate to ensure solubility and bioavailability.

Experimental Protocol

Following pre-treatment with *V. persicifolia* hexane extracts, zebrafish were exposed to diazepam or sodium valproate.

Key seizure parameters were recorded, including:

- Convulsion latency
- Seizure severity
- Survival rates

This protocol was adapted from [7,5].

5. Statistical Analysis

The statistical analysis of the data was carried out using comprehensive methods to ensure the robustness and reliability of the results. The selection of statistical tests was based on normality (Shapiro-Wilk test) and homoscedasticity (Levene's test). Depending on the assumptions met, the appropriate multiple comparison test was chosen. Statistical analysis was conducted using SPSS v29.0.2.0.

To enhance clarity, the analysis has been divided into three experimental phases, consistent with the structure of the main article:

1. Partition Method
2. Sequential Method
3. Co-administration with Pharmacological Controls

5.1. Partition Method

A one-way ANOVA was performed to assess the effects of different extract fractions obtained from the partition method on the PTZ-induced seizure model in zebrafish (*Danio rerio*).

5.1.1. Normality and Homoscedasticity Testing

(1) Shapiro-Wilk test was used to check for normality.
(2) Levene's test assessed homoscedasticity (equal variance) among groups.
(3) Even though some groups did not meet normality, the assumption of homoscedasticity was met, making one-way ANOVA robust to these violations.
(4) Tukey HSD for one-way ANOVA test was used as a post-hoc test.

Table S1. Summary statistics of results obtained using the partition method, including normality (Shapiro-Wilk test), homoscedasticity (Levene's test), and one-way ANOVA results.

Variable	Group	Mean	Median	SD	Variance	Max	Min	Shapiro-Wilks	Levene	ANOVA
**Latency Speed Index**	Control	162.43	162	24.609	605.619	205	129	0.949	0.11	< .001**
	**Hx 1**	160.83	149.5	52.826	2790.567	243	111	0.419		

Variable	Group	Mean	Median	SD	Variance	Max	Min	Shapiro-Wilks	Levene	ANOVA
**(Seconds)**	**Hx 10**	183.33	181.5	67.39	4541.467	303	106	0.419		
	**Hx 100**	260.8	269	55.818	3115.7	314	167	0.199		
	**Hx 1000**	226.6	226	44.925	2018.3	295	173	0.816		
	**DCM 1**	122.17	118	35.04	1227.767	182	89	0.365		
	**DCM 10**	85.5	81	24.44	597.5	132	60	0.092		
	**DCM 100**	198.33	134.5	190.09	36442.667	584	72	0.001*		
	**DCM 1000**	257.83	257	96.361	9285.367	419	125	0.708		
	**EtOAc 1**	112.5	109.5	28.62	819.1	159	81	0.655		
	**EtOAc 10**	155.17	159.5	25.888	670.167	191	117	0.96		
	**EtOAc 100**	96.5	95	15.228	231.9	120	76	0.975		
	**EtOAc 1000**	54.5	43.5	35.467	1257.9	122	29	0.034*		
	**MeOH 1**	173.17	155	52.678	2774.967	249	124	0.221		
	**MeOH 10**	143.5	138	37.383	1397.5	198	104	0.503		
	**MeOH 100**	149.67	157	26.372	695.467	176	104	0.388		
	**MeOH 1000**	181.67	183	18.468	341.067	201	160	0.231		
	**Ac 1**	158.33	157.5	36.291	1317.067	204	110	0.814		
	**Ac10**	131.5	147.5	37.453	1402.7	167	64	0.127		
	**Ac 100**	137.17	134	24.457	598.167	183	115	0.118		
	**Ac 1000**	144.67	146.5	47.781	2281.067	210	75	0.998		
**Whirpool Latency (Seconds)**	Control	195.29	189	43.976	1933.905	287	145	0.049*	0.399	< .001**
	**Hx 1**	173.17	156	53.383	2849.767	248	115	0.395		
	**Hx 10**	201	187.5	71.49	5110.8	329	110	0.263		
	**Hx 100**	278.4	273	59.496	3539.8	359	205	0.987		
	**Hx 1000**	286.2	288	73.754	5439.7	373	187	0.938		
	**DCM 1**	137.33	126.5	45.456	2066.267	217	96	0.323		
	**DCM 10**	109.67	102.5	31.494	991.867	163	77	0.463		
	**DCM 100**	223	152.5	193.48	37435.2	608	80	0.007*		

Variable	Group	Mean	Median	SD	Variance	Max	Min	Shapiro-Wilks	Levene	ANOVA
	**DCM 1000**	359.67	340	77.912	6070.267	460	269	0.409		
	**EtOAc 1**	151.67	149	44.996	2024.667	226	96	0.851		
	**EtOAc 10**	182.17	182	31.815	1012.167	222	142	0.571		
	**EtOAc 100**	122.17	118	26.514	702.967	169	97	0.272		
	**EtOAc 1000**	84	64.5	46.5	2148.8	159	36	0.288		
	**MeOH 1**	185.81	181	53.244	2834.967	263	127	0.695		
	**MeOH 10**	161.17	164.5	34.557	1194.167	204	108	0.816		
	**MeOH 100**	161.67	158	17.386	302.267	183	140	0.486		
	**MeOH 1000**	194.67	192.5	16.476	271.467	218	179	0.14		
	**Ac 1**	222	210.5	105.84	11202.8	417	124	0.169		
	**Ac10**	159.83	163.5	34.643	1200.167	216	122	0.367		
	**Ac 100**	154.67	136	39.883	1590.667	218	121	0.109		
	**Ac 1000**	168.33	179.5	51.999	2703.867	220	89	0.501		
**Posture loss latency (Seconds)**	Control	207	200	47.504	2256.667	307	153	0.031*	0.572	< .001**
	**Hx 1**	219.67	195.5	101.85	10374.667	398	127	0.253		
	**Hx 10**	209.5	197.5	72.241	5218.7	337	115	0.317		
	**Hx 100**	292.4	279	46.215	2135.8	354	241	0.734		
	**Hx 1000**	299	297	51.269	2628.5	384	258	0.119		
	**DCM 1**	154.33	143	35.517	1261.467	2211	123	0.125		
	**DCM 10**	166.17	158.5	55.944	3129.767	244	98	0.821		
	**DCM 100**	363.33	243.5	285.00	81230.267	887	140	0.071		
	**DCM 1000**	402.33	356.5	132.51	17559.467	636	290	0.174		
	**EtOAc 1**	185	180	33.335	1111.2	243	143	0.623		
	**EtOAc 10**	206.67	206.5	26.5	727.867	244	162	0.716		
	**EtOAc 100**	142.17	126.5	40.047	1603.767	206	100	0.361		
	**EtOAc 1000**	138.5	93.5	100.59	10119.5	283	50	0.082		

Variable	Group	Mean	Median	SD	Variance	Max	Min	Shapiro-Wilks	Levene	ANOVA
	**MeOH 1**	206.5	186.5	62.931	3960.3	296	139	0.369		
	**MeOH 10**	167.33	158	36.015	1297.067	233	122	0.774		
	**MeOH 100**	184.67	176.5	28.338	803.067	226	150	0.561		
	**MeOH 1000**	268.83	240	67.966	4619.367	355	212	0.039		
	**Ac 1**	321.5	299	106.94	11437.9	453	209	0.226		
	**Ac10**	227	210	91.056	8291.2	389	128	0.486		
	**Ac 100**	208	213	32.997	1088.8	242	162	0.45		
	**Ac 1000**	186.83	188	46.731	2183.767	205	136	0.524		

Abbreviations, SD: Standard deviation, Hx: Hexane, DM: Dichloromethane, Aet: Ethyl Acetate, Met: Methanol, Acu: Water.
*Group that does not meet the assumption of normality by the Shapiro-Wilks test.
**Statistically significant p value in the ANOVA test

Table S2. Detailed statistical results for the one-way ANOVA test applied to the partition method.

Variable	Comparison	Sum of squares	Degrees of freedom	Quadratic mean	F	P value
**Latency Speed Index (Seconds)**	Between groups	302237.85	20	15111.89	4.276	**<0.01***
	Within groups	367541.71	104	3534.06		
	Total	669779.57	124			
**Whirpool Latency (Seconds)**	Between groups	455385.67	20	22769.284	5.192	**<0.01***
	Within groups	456087.768	104	43.85.45		
	Total	911472.77	124			
**Posture loss latency (Seconds)**	Between groups	615541.45	20	30777.072	3.71	**<0.01***
	Within groups	862687.87	104	8295.08		
	Total	1478229.31	124			

*Statistically significant p value in the ANOVA test

5.1.2. Post-Hoc Analysis

To determine significant pairwise differences, a Tukey’s Honest Significant Difference (HSD) post-hoc test was conducted. This test controls the family-wise error rate, ensuring that multiple comparisons maintain statistical reliability.

Statistical Significance

- A p-value < 0.05 was considered statistically significant.
- Values between identical groups (e.g., control vs. control) were not shown, as they do not meet the assumption of independence. These cases were marked as "Not Applicable" in tables.
- To avoid redundancy, pairwise p-values were reported only once

Table S3. Tukey HSD post-hoc test: Pairwise p-values for Latency Speed Index in the one-way ANOVA.

Group	Control	Hx 1	Hx 10	Hx 100	Hx 1000	DCM 1	DCM 10	DCM 100	DCM 1000	EtOAc 1	EtOAc 10	EtOAc 100	EtOAc 1000	MeOH 1	MeOH 10	MeOH 100	MeOH 1000	Ac 1	Ac10	Ac 100	Ac1000
**Control**	NA	0.99	0.37	0.96	0.99	0.73	0.99	0.34	0.99	0.99	0.91	0.15	0.99	0.99	0.99	0.99	0.99	0.99	0.99	0.99	0.99
**Hx 1**		NA	0.99	0.41	0.96	0.99	0.82	0.99	0.37	0.99	0.99	0.95	0.22	0.99	0.99	0.99	0.99	0.99	0.99	0.99	0.99
**Hx 10**			NA	0.84	0.99	0.97	0.36	0.99	0.83	0.88	0.99	0.59	0.04*	0.99	0.99	0.99	0.99	0.99	0.99	0.99	0.99
**Hx 100**				NA	0.99	0.03*	<0.01*	0.98	0.99	0.03*	0.31	<0.01*	<0.01*	0.66	0.15	0.22	0.81	0.36	0.06	0.09	0.16
**Hx 1000**					NA	0.33	0.02*	0.99	0.99	0.19	0.91	0.06	<0.01*	0.99	0.75	0.85	0.99	0.94	0.5	0.62	0.77
**DCM 1**						NA	0.99	0.8	0.02*	0.99	0.99	0.99	0.92	0.99	0.99	0.92	0.99	0.99	0.99	0.99	0.99
**DCM 10**							NA	0.73	<0.01*	0.99	0.9	0.99	0.99	0.57	0.98	0.95	0.39	0.86	0.99	0.99	0.98
**DCM 100**								NA	0.98	0.61	0.99	0.29	<0.01*	0.99	0.99	0.99	0.99	0.99	0.93	0.97	0.99
**DCM 1000**									NA	<0.01*	0.27	<0.01*	<0.01*	0.64	0.13	0.19	0.8	0.33	0.04*	0.07	0.14
**EtOAc 1**										NA	0.99	0.99	0.98	0.97	0.99	0.99	0.9	0.99	0.99	0.99	0.99
**EtOAc 10**											NA	0.98	0.31	0.99	0.99	0.99	0.99	0.99	0.99	0.99	0.99
**EtOAc 100**												NA	0.99	0.79	0.99	0.99	0.62	0.96	0.99	0.99	0.99
**EtOAc 1000**													NA	0.09	0.54	0.41	0.04*	0.25	0.79	0.68	0.52
**MeOH 1**														NA	0.99	0.99	0.99	0.99	0.99	0.99	0.99
**MeOH 10**															NA	0.99	0.99	0.99	0.99	0.99	0.99
**MeOH 100**																NA	0.99	0.99	0.99	0.99	0.99
**MeOH 1000**																	NA	0.99	0.99	0.99	0.99
**Ac 1**																		NA	0.99	0.99	0.99
**Ac10**																			NA	0.99	0.99
**Ac 100**																				NA	0.99
**Ac 1000**																					NA

Abbreviations, SD: Standard deviation, Hx: Hexane, DCM: Dichloromethane, EtOAc: Ethyl Acetate, MeOH: Methanol, Ac: Water. NA: not applicable.
*Statistically significant p value in the ANOVA test

Table S4. Tukey HSD post-hoc test: Pairwise p-values for Whirlpool Latency in the one-way ANOVA.

Group	Control	Hx 1	Hx 10	Hx 100	Hx 1000	DCM 1	DCM 10	DCM 100	DCM 1000	EtOAc 1	EtOAc 10	EtOAc 100	EtOAc 1000	MeOH 1	MeOH 10	MeOH 100	MeOH 1000	Ac 1	Ac10	Ac 100	Ac1000
**Control**	NA	0.99	0.99	0.84	0.72	0.99	0.74	0.99	<0.01*	0.99	0.99	0.91	0.26	0.99	0.99	0.99	0.99	0.99	0.99	0.99	0.99
**Hx 1**		NA	0.99	0.52	0.38	0.99	0.99	0.99	<0.01*	0.99	0.99	0.99	0.73	0.99	0.99	0.99	0.99	0.99	0.99	0.99	0.99
**Hx 10**			NA	0.93	0.85	0.98	0.69	0.99	<0.01*	0.99	0.99	<0.01*	0.24	0.99	0.99	0.99	0.99	0.99	0.99	0.99	0.99
**Hx 100**				NA	0.99	0.08	<0.01*	0.99	0.9	0.19	0.68	0.02*	<0.01*	0.75	9.31	0.32	0.87	0.99	0.29	0.22	0.43
**Hx 1000**					NA	0.04*	<0.01*	0.99	0.96	0.12	0.54	0.01*	<0.01*	0.61	0.21	0.76	0.76	0.99	0.19	0.14	0.3
**DCM 1**						NA	0.99	0.79	<0.01*	0.99	0.99	0.99	0.99	0.99	0.99	0.99	0.99	0.81	0.99	0.99	0.99
**DCM 10**							NA	0.29	<0.01*	0.99	0.94	0.99	0.99	0.91	0.99	0.99	0.8	0.3	0.99	0.99	0.99
**DCM 100**								NA	0.07	0.95	0.99	0.51	0.05	0.99	0.98	0.99	0.99	0.99	0.99	0.97	0.99
**DCM 1000**									NA	<0.01*	<0.01*	<0.01*	<0.01*	<0.01*	<0.01*	<0.01*	<0.01*	0.06	<0.01*	<0.01*	<0.01*
**EtOAc 1**										NA	0.99	0.99	0.97	0.99	0.99	0.99	0.99	0.96	0.99	0.99	0.99
**EtOAc 10**											NA	0.99	0.56	0.99	0.99	0.99	0.99	0.99	0.99	0.99	0.99
**EtOAc 100**												NA	0.99	0.98	0.99	0.99	0.94	0.53	0.99	0.99	0.99
**EtOAc 1000**													NA	0.49	0.9	0.9	0.33	0.06	0.91	0.95	0.81
**MeOH 1**														NA	0.99	0.99	0.99	0.99	0.99	0.99	0.99
**MeOH 10**															NA	0.99	0.99	0.99	0.99	0.99	0.99
**MeOH 100**																NA	0.99	0.99	0.99	0.99	0.99
**MeOH 1000**																	NA	0.99	0.99	0.99	0.99
**Ac 1**																		NA	0.98	0.97	0.99
**Ac10**																			NA	0.99	0.99
**Ac 100**																				NA	0.99
**Ac 1000**																					NA

Abbreviations, SD: Standard deviation, Hx: Hexane, DCM: Dichloromethane, EtOAc: Ethyl Acetate, MeOH: Methanol, Ac: Water. NA: not applicable.
*Statistically significant p value in the ANOVA test

Table S5. Tukey HSD post-hoc test: Pairwise **p-values for Posture Loss Latency** in the one-way ANOVA.

Group	Control	Hx 1	Hx 10	Hx 100	Hx 1000	DCM 1	DCM 10	DCM 100	DCM 1000	EtOAc 1	EtOAc 10	EtOAc 100	EtOAc 1000	MeOH 1	MeOH 10	MeOH 100	MeOH 1000	Ac 1	Ac10	Ac 100	Ac1000
Control	NA	0.99	0.98	0.99	0.97	0.99	0.99	0.22	0.03*	0.99	0.99	0.99	0.99	0.99	0.99	0.99	0.99	0.77	0.99	0.99	0.99
Hx 1		NA	0.99	0.99	0.99	0.99	0.99	0.44	0.09	0.99	0.99	0.99	0.99	0.99	0.99	0.99	0.99	0.93	0.99	0.99	0.99
Hx 10			NA	0.99	0.99	0.99	0.99	0.31	0.05	0.99	0.99	0.99	0.99	0.99	0.99	0.99	0.99	0.85	0.99	0.99	0.99
Hx 100				NA	0.99	0.61	0.76	0.99	0.91	0.93	0.99	0.44	0.4	0.99	0.77	0.93	0.99	0.99	0.99	0.99	0.94
Hx 1000					NA	0.52	0.,68	0.99	0.95	0.88	0.98	0.36	0.32	0.98	0.69	0.88	0.99	0.99	0.99	0.98	0.89
DCM 1						NA	0.99	0.02*	<0.01*	0.99	0.99	0.99	0.99	0.99	0.99	0.99	0.83	0.18	0.99	0.99	0.99
DCM 10							NA	0.04*	<0.01*	0.99	0.99	0.99	0.99	0.99	0.99	0.99	0.92	0.29	0.99	0.99	0.99
DCM 100								NA	0.99	0.11	0.28	<0.01*	<0.01*	0.27	0.04*	0.11	0.96	0.99	0.54	0.29	0.12
DCM 1000									NA	0.01*	0.04*	<0.01*	<0.01*	0.04*	<0.01*	0.01*	0.58	0.99	0.12	0.04*	0.01*
EtOAc 1										NA	0.99	0.99	0.99	0.99	0.99	0.99	0.99	0.54	0.99	0.99	0.99
EtOAc 10											NA	0.99	0.99	0.99	0.99	0.99	0.99	0.82	0.99	0.99	0.99
EtOAc 100												NA	0.99	0.99	0.99	0.99	0.67	0.1	0.98	0.99	0.99
EtOAc 1000													NA	0.99	0.99	0.99	0.62	0.08	0.98	0.99	0.99
MeOH 1														NA	0.99	0.99	0.99	0.82	0.99	0.99	0.99
MeOH 10															NA	0.99	0.93	0.31	0.99	0.99	0.99
MeOH 100																NA	0.99	0.53	0.99	0.99	0.99
MeOH 1000																	NA	0.99	0.99	0.99	0.99
Ac 1																		NA	0.96	0.84	0.57
Ac10																			NA	0.99	0.99
Ac 100																				NA	0.99
Ac 1000																					NA

Abbreviations, SD: Standard deviation, Hx: Hexane, DCM: Dichloromethane, EtOAc: Ethyl Acetate, MeOH: Methanol, Ac: Water. NA: not applicable.
*Statistically significant p value in the ANOVA test

5.2. Sequential Method

A one-way ANOVA was conducted to evaluate the effects of different treatments obtained through the sequential extraction method on the PTZ-induced seizure model in zebrafish (*Danio rerio*).

5.2.1. Normality and Homoscedasticity Testing

(1) Shapiro-Wilk test was used to assess normality of data distribution.
(2) Levene's test determined homoscedasticity (equal variance) among experimental groups.
(3) Some groups did not meet normality assumptions but satisfied homoscedasticity requirements, making ANOVA a robust statistical test under these conditions.

Table S6. Summary statistics of the results obtained using the **sequential extraction method**, including:

Variable	Group	Mean	Median	SD	Variance	Max	Min	Shapiro-Wilks	Levene	ANOVA
**Latency Speed Index (Seconds)**	Control	162.43	162	24.609	605.619	205	129	0.95	0.44	< .001**
	**Hx 1**	92.17	77	56.262	3165.367	196	39	0.22		
	**Hx 10**	66	61	30.666	940.4	119	39	0.21		
	**Hx 100**	138.67	125.5	75.736	5735.867	262	67	0.33		
	**Hx 1000**	303.83	308.81	75.93	5765.367	410	216	0.69		
	**DCM 1**	166.83	183	34.713	1204.967	200	120	0.08		
	**DCM 10**	177.33	186	40.893	1672.267	223	100	0.06		
	**DCM 100**	162.67	168.5	59.762	3571.467	226	79	0.47		
	**DCM 1000**	174.83	169.5	38.452	1478.567	235	123	0.97		
	**EtOAc 1**	140.83	162	78.219	6118.167	234	26	0.62		
	**EtOAc 10**	72.83	66	34.505	1190.567	123	22	0.78		
	**EtOAc 100**	334.67	328.5	105.84	11202.67	469	190	0.83		
	**EtOAc 1000**	486.67	298	333.57	111265.07	977	248	0.01*		
	**MeOH 1**	228.83	240	48.697	2371.37	280	155	0.54		
	**MeOH 10**	246	248	27.655	764.8	278	206	0.79		
	**MeOH 100**	150.67	153.5	59.399	3528.27	233	80	0.72		
	**MeOH 1000**	155	142.5	28.81	830	195	125	0.16		
**Whirpool Latency (Seconds)**	Control	195.29	189	43.976	1933.905	287	145	0.05	0.96	< .001**
	**Hx 1**	141	102	98.908	9782.8	282	44	0.18		
	**Hx 10**	84	64	50.291	2529.2	163	42	0.15		
	**Hx 100**	316.5	192.5	351.93	123856.7	997	69	0.02*		
	**Hx 1000**	448.5	447	131.28	17233.5	595	266	0.63		
	**DCM 1**	244.67	232	92.21	8501.867	395	142	0.56		
	**DCM 10**	204.83	188.5	66.26	4390.167	288	108	0.42		
	**DCM 100**	241.5	255	68.86	4742.3	325	120	0.55		
	**DCM 1000**	198.17	198	50.95	2595.367	263	126	0.94		
	**EtOAc 1**	178.83	195	92.77	8606.967	300	59	0.74		
	**EtOAc 10**	90.5	85	23.02	529.9	124	63	0.75		
	**EtOAc 100**	892.33	829.5	553.84	306741.467	1800	325	0.48		
	**EtOAc 1000**	732.33	546	562.05	314773.867	1800	315	0.04*		

Variable	Group	Mean	Median	SD	Variance	Max	Min	Shapiro-Wilks	Levene	ANOVA
	**MeOH 1**	261.83	242.5	97.95	9594.167	444	165	0.21		
	**MeOH 10**	285	278	56.54	3196.4	385	209	0.15		
	**MeOH 100**	155.83	161.5	59.01	3482.167	235	83	0.62		
	**MeOH 1000**	173.67	173.5	31.17	971.467	210	128	0.65		
**Posture loss latency (Seconds)**	Control	207	200	47.5	2256.667	307	154	0.03*	0.57	< .001**
	**Hx 1**	148.33	109.5	101.05	10210.267	291	49	0.17		
	**Hx 10**	101.67	91.5	51.68	2670.267	190	49	0.46		
	**Hx 100**	328.33	201	352.7	124411.467	1005	74	0.03*		
	**Hx 1000**	479.33	480	134.46	18078.667	635	269	0.82		
	**DCM 1**	302	283	112.91	12748.4	495	196	0.35		
	**DCM 10**	211.17	193	68.56	4700.967	301	113	0.45		
	**DCM 100**	277	270	98.28	9658.8	422	128	0.96		
	**DCM 1000**	283.33	249	136.45	18619.467	493	130	0.70		
	**EtOAc 1**	234.17	244.5	67.9	4611.767	305	114	0.44		
	**EtOAc 10**	118.33	113.5	41.26	1702.267	193	79	0.22		
	**EtOAc 100**	1125.17	1012.5	589.8	347877.767	1800	296	0.46		
	**EtOAc 1000**	780.83	549.5	521.5	271934.167	1800	458	0.01*		
	**MeOH 1**	304.83	261.5	105.5	11132.567	449	204	0.12		
	**MeOH 10**	332.5	320.5	90.11	8119.5	467	214	0.95		
	**MeOH 100**	164.33	165	65.07	4233.867	259	87	0.72		
	**MeOH 1000**	218	214	70.56	4978	345	131	0.25		

Abbreviations, SD: Standard deviation, Hx: Hexane, DCM: Dichloromethane, EtOAc: Ethyl Acetate, MeOH: Methanol.
*Group that does not meet the assumption of normality by the Shapiro-Wilks test.
**Statistically significant p value in the ANOVA test

Table S7. Detailed statistics of the one-way ANOVA test conducted for the sequential extraction method.

Variable	Comparison	Sum of squares	Degrees of freedom	Quadratic mean	F	P value
**Latency Speed Index (Seconds)**	Between groups	1053386	16	65836.64	7.01	**<0.01***
	Within groups	807659.5	86	9391.39		
	Total	1861046	102			
**Whirpool Latency (Seconds)**	Between groups	4580414	16	286275.9	5.977	**<0.01***
	Within groups	4119245	86	47898.2		
	Total	8699659	102			
**Posture loss latency (Seconds)**	Between groups	6450778	16	403173.6	8.079	**<0.01***
	Within groups	4291981	86	49906.76		
	Total	10742759	102			

*Statistically significant p value in the ANOVA test

5.2.2. Post-Hoc Analysis

To identify statistically significant differences between treatment groups, Tukey's Honest Significant Difference (HSD) post-hoc test was used. This test controls for the family-wise error rate, ensuring that pairwise comparisons are statistically reliable.

Significance Criteria

- A p-value < 0.05 was considered statistically significant.
- Values between identical groups (e.g., control vs. control) were not shown, as they do not meet the assumption of independence. These cases were marked as "Not Applicable" in tables.
- To avoid redundancy, pairwise p-values were reported only once

Table S8. Tukey HSD post-hoc test results: Pairwise p-values for Latency Speed Index in the one-way ANOVA.

Group	Control	Hx 1	Hx 10	Hx 100	Hx 1000	DCM 1	DCM 10	DCM 100	DCM 1000	EtOAc 1	EtOAc 10	EtOAc 100	EtOAc 1000	MeOH 1	MeOH 10	MeOH 100	MeOH 1000
**Control**	N/A	0.99	0.93	0.99	0.42	0.99	0.99	0.99	0.99	0.99	0.96	0.13	0.99	0.99	0.98	0.99	0.99
**Hx 1**		N/A	0.99	0.99	0.03*	0.99	0.98	0.99	0.99	0.99	0.99	<0.01*	<0.01*	0.55	0.34	0.99	0.99
**Hx 10**			N/A	0.99	<0.01*	0.92	0.85	0.95	0.87	0.99	0.99	<0.01*	<0.01*	0.25	0.13	0.98	0.97
**Hx 100**				N/A	0.23	0.99	0.99	0.99	0.99	0.99	0.99	0.06	<0.01*	0.97	0.88	0.99	0.99
**Hx 1000**					N/A	0.55	0.68	0.49	0.65	0.25	<0.01	0.99	0.11	0.99	0.99	0.35	0.39
**DCM 1**						N/A	0.99	0.99	0.99	0.99	0.96	0.21	<0.01*	0.99	0.99	0.99	0.99
**DCM 10**							N/A	0.99	0.99	0.99	0.9	0.3	<0.01*	0.99	0.99	0.99	0.99
**DCM 100**								N/A	0.99	0.99	0.97	0.18	<0.01*	0.99	0.99	0.99	0.99
**DCM 1000**									N/A	0.99	0.92	0.28	<0.01*	0.99	0.99	0.99	0.99
**EtOAc 1**										N/A	0.99	0.06	<0.01*	0.97	0.89	0.99	0.99
**EtOAc 10**											N/A	<0.01*	<0.01*	0.32	0.17	0.99	0.99
**EtOAc 100**												N/A	0.36	0.89	0.97	0.11	0.13
**EtOAc 1000**													N/A	<0.01*	<0.01*	<0.01*	<0.01*
**MeOH 1**														N/A	0.99	0.99	0.99
**MeOH 10**															N/A	0.95	0.97
**MeOH 100**																N/A	0.99
**MeOH 1000**																	N/A

Abbreviations, SD: Standard deviation, Hx: Hexane, DCM: Dichloromethane, EtOAc: Ethyl Acetate, MeOH: Methanol. NA: not applicable.
*Statistically significant p value in the ANOVA test

Table S9. Tukey HSD post-hoc test results: Pairwise p-values for Whirlpool Latency in the one-way ANOVA.

Group	Control	Hx 1	Hx 10	Hx 100	Hx 1000	DCM 1	DCM 10	DCM 100	DCM 1000	EtOAc 1	EtOAc 10	EtOAc 100	EtOAc 1000	MeOH 1	MeOH 10	MeOH 100	MeOH 1000
**Control**	N/A	0.99	0.99	0.99	0.8	0.99	0.99	0.99	0.99	0.99	0.99	<0.01*	<0.01*	0.99	0.99	0.99	0.99
**Hx 1**		N/A	0.99	0.99	0.56	0.99	0.99	0.99	0.99	0.99	0.99	<0.01*	<0.01*	0.99	0.99	0.99	0.99
**Hx 10**			N/A	0.91	0.26	0.99	0.99	0.99	0.99	0.99	0.99	<0.01*	<0.01*	0.99	0.97	0.99	0.99
**Hx 100**				N/A	0.99	0.99	0.99	0.99	0.99	0.99	0.92	<0.01*	0.11	0.99	0.99	0.99	0.99
**Hx 1000**					N/A	0.97	0.88	0.96	0.85	0.76	0.29	0.06	0.69	0.98	0.99	0.64	0.74
**DCM 1**						N/A	0.99	0.99	0.99	0.99	0.99	<0.01*	0.02*	0.99	0.99	0.99	0.99
**DCM 10**							N/A	0.99	0.99	0.99	0.99	<0.01*	<0.01*	0.99	0.99	0.99	0.99
**DCM 100**								N/A	0.99	0.99	0.99	<0.01*	0.02*	0.99	0.99	0.99	0.99
**DCM 1000**									N/A	0.99	0.99	<0.01*	<0.01*	0.99	0.99	0.99	0.99
**EtOAc 1**										N/A	0.99	<0.01*	<0.01*	0.99	0.99	0.99	0.99
**EtOAc 10**											N/A	<0.01*	<0.01*	0.99	0.98	0.99	0.99
**EtOAc 100**												N/A	0.99	<0.01*	<0.01*	<0.01*	<0.01*
**EtOAc 1000**													N/A	0.03*	0.05	<0.01*	<0.01*
**MeOH 1**														N/A	0.99	0.99	0.99
**MeOH 10**															N/A	0.99	0.99
**MeOH 100**																N/A	0.99
**MeOH 1000**																	N/A

Abbreviations, SD: Standard deviation, Hx: Hexane, DM: Dichloromethane, Aet: Ethyl Acetate, Met: Methanol. NA: not applicable.
*Statistically significant p value in the ANOVA test

Table S10. Tukey HSD post-hoc test results: Pairwise p-values for Posture Loss Latency in the one-way ANOVA.

Group	Control	Hx 1	Hx 10	Hx 100	Hx 1000	DCM 1	DCM 10	DCM 100	DCM 1000	EtOAc 1	EtOAc 10	EtOAc 100	EtOAc 1000	MeOH 1	MeOH 10	MeOH 100	MeOH 1000
**Control**	N/A	0.99	0.99	0.99	0.73	0.99	0.99	0.99	0.99	0.99	0.99	<.0.01*	<.0.01*	0.99	0.99	0.99	0.99
**Hx 1**		N/A	0.99	0.99	0.46	0.99	0.99	0.99	0.99	0.99	0.99	<.0.01*	<.0.01*	0.99	0.99	0.99	0.99
**Hx 10**			N/A	0.94	0.24	0.98	0.99	0.99	0.99	0.99	0.99	<.0.01*	<.0.01*	0.97	0.93	0.99	0.99
**Hx 100**				N/A	0.99	0.99	0.99	0.99	0.99	0.99	0.97	<.0.01*	0.06	0.99	0.99	0.99	0.99
**Hx 1000**					N/A	0.99	0.79	0.98	0.98	0.88	0.31	<.0.01*	0.63	0.99	0.99	0.55	0.83
**DCM 1**						N/A	0.99	0.99	0.99	0.99	0.99	<.0.01*	0.03*	0.99	0.99	0.99	0.99
**DCM 10**							N/A	0.99	0.99	0.99	0.99	<.0.01*	<.0.01*	0.99	0.99	0.99	0.99
**DCM 100**								N/A	0.99	0.99	0.99	<.0.01*	<.0.01*	0.99	0.99	0.99	0.99
**DCM 1000**									N/A	0.99	0.99	<.0.01*	0.02*	0.99	0.99	0.99	0.99
**EtOAc 1**										N/A	0.99	<.0.01*	<.0.01*	0.99	0.99	0.99	0.99
**EtOAc 10**											N/A	<.0.01*	<.0.01*	0.98	0.96	0.99	0.99
**EtOAc 100**												N/A	0.39	<.0.01*	<.0.01*	<.0.01*	<.0.01*
**EtOAc 1000**													N/A	0.03*	0.06	<.0.01*	<.0.01*
**MeOH 1**														N/A	0.99	0.99	0.99
**MeOH 10**															N/A	0.99	0.99
**MeOH 100**																N/A	0.99
**MeOH 1000**																	N/A

Abbreviations, SD: Standard deviation, Hx: Hexane, DM: Dichloromethane, Aet: Ethyl Acetate, Met: Methanol. NA: not applicable.
*Statistically significant p value in the ANOVA test

5.3. Co-administration with Pharmacological Controls

To evaluate the effects of different treatments on the PTZ-induced seizure model in zebrafish (*Danio rerio*), various statistical tests were applied depending on the assumptions of normality and homoscedasticity.

The treatments in this phase included the addition of pharmacological controls (diazepam and sodium valproate) in combination with *V. persicifolia* extracts.

One-way ANOVA was used for Latency Speed Index, as some groups did not meet normality assumptions but satisfied homoscedasticity, making ANOVA robust to these violations.

Kruskal-Wallis test was applied for Whirlpool Latency and Posture Loss Latency, as these datasets did not meet homoscedasticity assumptions, requiring a non-parametric approach.

5.3.1. Normality and Homoscedasticity Testing

(1) Shapiro-Wilk test assessed normality of data distribution.

(2) Levene's test determined homoscedasticity (equal variance) across groups.

(3) Based on these results:

a) One-way ANOVA was applied to Latency Speed Index.

b) Kruskal-Wallis test was applied to Whirlpool Latency and Posture Loss Latency.

Table S11. Summary statistics for the results obtained from the co-administration study, including: Normality (Shapiro-Wilk test); Homoscedasticity (Levene's test); Statistical results from One-way ANOVA and Kruskal-Wallis tests

Variable	Group	Mean	Median	SD	Variance	Max	Min	Shapiro-Wilks	Levene	ANOVA or Kruskall Walls *p value*
**Latency Speed Index (Seconds)**	Control	162.43	162	24.61	605.619	205	129	0.949	0.3	0.12[a]
	DZP 75μM	254.83	231	76.36	5830.567	357	166	0.408		
	Val-Na 75μM	247.83	244.5	34.02	1157.367	288	196	0.737		
	Hx 5μM	257.67	266	80.37	6459.067	366	121	0.675		
	Hx 10μM	247.17	217.5	66.76	4456.567	356	192	0.091		
	Hx 15μM	219	220.5	56.73	3218.4	307	148	0.909		
	Co-Ad Val-Na+Hx	272.33	213	133.28	17764.667	537	193	0.003*		
	Co-Ad Dzp+Hx	203.17	199	15.198	230.967	233	192	0.018*		
**Whirpool Latency (Seconds)**	Control	195.29	189	43.976	1933.905	287	145	0.049*	<0.01**	< .001[b]***
	DZP 75μM	772.83	582	416.4	173392.567	1593	529	0.004*		
	Val-Na 75μM	312.33	304	78.95	6233.867	444	217	0.833		
	Hx 5μM	506.33	544.5	253.47	64243.467	862	196	0.681		
	Hx 10μM	442.17	338.5	265.52	70502.967	919	211	0.137		
	Hx 15μM	724.67	829	297.62	88578.267	958	174	0.081		
	Co-Ad Val-Na+Hx	343.17	298	126.39	15975.367	551	222	0.305		

Variable	Group	Mean	Median	SD	Variance	Max	Min	Shapiro-Wilks	Levene	ANOVA or Kruskall Walls *p value*
	Co-Ad Dzp+Hx	1128.3	1236	709.25	503041.067	1800	266	0.109		
**Posture loss latency (Seconds)**	Control	207	200	47.504	2256.667	307	154	0.031*	<0.01**	< .001[b]***
	DZP 75μM	800.83	614.5	408.23	166648.567	1597	536	0.009*		
	Val-Na 75μM	711.33	815	210.73	44406.667	865	380	0.056		
	Hx 5μM	625	492	407.45	1660013.6	1325	274	0.239		
	Hx 10μM	653.33	547.5	428.14	183303.467	1325	222	0.43		
	Hx 15μM	869.5	941.5	242.24	58681.1	1078	398	0.041*		
	Co-Ad Val-Na+Hx	401.17	396	115.72	13391.367	563	255	0.818		
	Co-Ad Dzp+Hx	903.67	712	693.23	480571.867	1800	250	0.161		

Abbreviations, SD: Standard deviation, Dzp: Diazepam, Val-Na: Sodium Valproate, Hx: Hexane, Coad: Co-administration.
[A]One-way ANOVA test, [b]Kruskall-Wallis test.
*Group that does not meet the assumption of normality by the Shapiro-Wilks test
**Group that does not meet the assumption of homoscedasticity by the Levene test
***Statistically significant p value in One-Way ANOVA or Kruskall-Wallis test.

Table S12. Detailed One-way ANOVA statistics for Latency Speed Index in the co-administration study.

Variable	Comparison	Sum of squares	Degrees of freedom	Quadratic mean	F	P value
**Latency Speed Index (Seconds)**	Between groups	59601.92	7	8514.56	1.75	**0.12**
	Within groups	199221.7	41	4859.066		
	Total	258823.6	48			

*Statistically significant p value in the ANOVA test

5.3.2. Post-Hoc Analysis

Latency Speed Index

Tukey's Honest Significant Difference (HSD) test was applied for post-hoc pairwise comparisons.

However, since ANOVA did not reveal statistical significance, no further Tukey HSD results were reported.

Whirlpool Latency & Posture Loss Latency

For these non-parametric datasets, Mann-Whitney U test with Bonferroni correction was conducted to compare pairs of treatment groups.

Bonferroni correction for Mann-Whitney U test was applied to account for multiple comparisons, using the formula:

$$p' = 1 - (1 - p)^c$$

Where c is the number of comparisons.

- Significant p-values ($p < 0.05$) were adjusted using this Bonferroni method.
- Values between identical groups (e.g., Control vs. Control) were marked as "Not Applicable", as they do not meet independence assumptions.
- To avoid redundancy, pairwise p-values were reported only once.

Table S13. Mann-Whitney U test with Bonferroni correction results: Pairwise p-values for Whirlpool Latency, as Kruskal-Wallis post-hoc analysis.

Group	Control	DZP 75µM	Val-Na 75µM	Hx 5µM	Hx 10µM	Hx 15µM	Co-Ad Val-Na+Hx	Co-Ad Dzp+Hx
Control	N/A	<0.01[a]*	0.13	0.10[a]	0.18[a]	<0.01[a]*	0.09	<0.01[a]*
DZP 75µM		N/A	0.35[a]	0.28	0.19	0.92	0.06	0.82
Val-Na 75µM			N/A	0.35	0.47	0.05	0.88	0.19[a]
Hx 5µM				N/A	0.83	0.32	0.44	0.19
Hx 10µM					N/A	0.23	0.57	0.13
Hx 15µM						N/A	0.08	0.74
Co-Ad Val-Na+Hx							N/A	0.29[a]
Co-Ad Dzp+Hx								N/A

Abbreviations, SD: Standard deviation, Dzp: Diazepam, Val-Na: Sodium Valproate, Hx: Hexane, Coad: Co-administration.
[A]p-value obtained from the Mann-Whitney U test, corrected by Bonferroni method
*Statistically significant p value

Table S14. Mann-Whitney U test with Bonferroni correction results: Pairwise p-values for Posture Loss Latency, as Kruskal-Wallis post-hoc analysis.

Group	Control	DZP 75µM	Val-Na 75µM	Hx 5µM	Hx 10µM	Hx 15µM	Co-Ad Val-Na+Hx	Co-Ad Dzp+Hx
Control	N/A	<0.01[a]*	0.01[a]*	0.08[a]	0.07[a]	<0.01[a]*	0.09	0.01*
DZP 75µM		N/A	0.75	0.38	0.43	0.59	0.08	0.76
Val-Na 75µM			N/A	0.58	0.64	0.39	0.16	0.98
Hx 5µM				N/A	0.94	0.16	0.39	0.57
Hx 10µM					N/A	0.19	0.35	0.62
Hx 15µM						N/A	0.19[a]	0.41
Co-Ad Val-Na+Hx							N/A	0.15
Co-Ad Dzp+Hx								N/A

Abbreviations, SD: Standard deviation, Dzp: Diazepam, Val-Na: Sodium Valproate, Hx: Hexane, Coad: Co-administration.
[A]p-value obtained from the Mann-Whitney U test, corrected by Bonferroni method
*Statistically significant p value

6. References

(1) UNAM. *Atlas de las Plantas de la Medicina Tradicional Mexicana. Huichín*. 2009. http://www.medicinatradicionalmexicana.unam.mx/apmtm/termino.php?l=3&t=huichin (accessed).

(2) Fonseca-Chávez, R. E., Rivera-Levario, L. A., Vázquez-García, L. . *Guía ilustrada de plantas medicinales en el Valle de México*; Instituto Nacional de los Pueblos Indígenas, 2020.

(3) Akinpelu, L. A.; Akanmu, M. A.; Obuotor, E. M. Mechanism of Anticonvulsant Effects of Ethanol Leaf Extract and Fractions of Milicia excelsa (Moraceae) in Mice. *Journal of Pharmaceutical Research International* **2018**, *23* (4), 1-11. DOI: 10.9734/jpri/2018/42430.

(4) Sulaimon, L. A.; Anise, E. O.; Obuotor, E. M.; Samuel, T. A.; Moshood, A. I.; Olajide, M.; Fatoke, T. In vitro antidiabetic potentials, antioxidant activities and phytochemical profile of african black pepper (Piper guineense). *Clinical Phytoscience* **2020**, *6* (1). DOI: 10.1186/s40816-020-00236-2.

(5) Mussulini, B. H.; Leite, C. E.; Zenki, K. C.; Moro, L.; Baggio, S.; Rico, E. P.; Rosemberg, D. B.; Dias, R. D.; Souza, T. M.; Calcagnotto, M. E.; et al. Seizures induced by pentylenetetrazole in the adult zebrafish: a detailed behavioral characterization. *PLoS One* **2013**, *8* (1), e54515. DOI: 10.1371/journal.pone.0054515.

(6) Almeida, E. R.; Lima-Rezende, C. A.; Schneider, S. E.; Garbinato, C.; Pedroso, J.; Decui, L.; Aguiar, G. P. S.; Muller, L. G.; Oliveira, J. V.; Siebel, A. M. Micronized Resveratrol Shows Anticonvulsant Properties in Pentylenetetrazole-Induced Seizure Model in Adult Zebrafish. *Neurochem Res* **2021**, *46* (2), 241-251. DOI: 10.1007/s11064-020-03158-0.

(7) Lee, Y.; Kim, D.; Kim, Y. H.; Lee, H.; Lee, C. J. Improvement of pentylenetetrazol-induced learning deficits by valproic acid in the adult zebrafish. *Eur J Pharmacol* **2010**, *643* (2-3), 225-231. DOI: 10.1016/j.ejphar.2010.06.041.
